# *Brucella* Rough Mutant Induce Macrophage Death via Activating IRE1α Pathway of Endoplasmic Reticulum Stress by Enhanced T4SS Secretion

**DOI:** 10.3389/fcimb.2017.00422

**Published:** 2017-09-27

**Authors:** Peng Li, Mingxing Tian, Yanqing Bao, Hai Hu, Jiameng Liu, Yi Yin, Chan Ding, Shaohui Wang, Shengqing Yu

**Affiliations:** ^1^Shanghai Veterinary Research Institute, Chinese Academy of Agricultural Sciences, Shanghai, China; ^2^Jiangsu Co-innovation Center for Prevention and Control of Important Animal Infectious Diseases and Zoonosis, Yangzhou, China

**Keywords:** *Brucella*, Type IV secretion system, lipopolysaccharide, VjbR, endoplasmic reticulum stress

## Abstract

*Brucella* is a Gram-negative facultative intracellular pathogen that causes the worldwide zoonosis, known as brucellosis. *Brucella* virulence relies mostly on its ability to invade and replicate within phagocytic cells. The type IV secretion system (T4SS) and lipopolysaccharide are two major *Brucella* virulence factors. *Brucella* rough mutants reportedly induce the death of infected macrophages, which is T4SS dependent. However, the underlying molecular mechanism remains unclear. In this study, the T4SS secretion capacities of *Brucella* rough mutant and its smooth wild-type strain were comparatively investigated, by constructing the firefly luciferase fused T4SS effector, BPE123 and VceC. In addition, quantitative real-time PCR and western blotting were used to analyze the T4SS expression. The results showed that T4SS expression and secretion were enhanced significantly in the *Brucella* rough mutant. We also found that the activity of the T4SS *virB* operon promoter was notably increased in the *Brucella* rough mutant, which depends on quorum sensing-related regulators of VjbR upregulation. Cell infection and cell death assays revealed that deletion of *vjbR* in the *Brucella* rough mutant absolutely abolished cytotoxicity within macrophages by downregulating T4SS expression. This suggests that up-regulation of T4SS promoted by VjbR in rough mutant Δ*rfbE* contribute to macrophage death. In addition, we found that the *Brucella* rough mutant induce macrophage death via activating IRE1α pathway of endoplasmic reticulum stress. Taken together, our study provide evidence that in comparison to the *Brucella* smooth wild-type strain, VjbR upregulation in the *Brucella* rough mutant increases transcription of the *virB* operon, resulting in overexpression of the *T4SS* gene, accompanied by the over-secretion of effecter proteins, thereby causing the death of infected macrophages via activating IRE1α pathway of endoplasmic reticulum stress, suggesting novel insights into the molecular mechanisms associated with *Brucella* rough mutant-induced macrophage cytotoxicity.

## Introduction

*Brucella* is a Gram-negative facultative intracellular bacterial species that causes zoonotic brucellosis, characterized by reproductive disease in domestic animals and chronic debilitating disease in humans (Boschiroli et al., 2001; Franco et al., 2007; Whatmore, 2009). Brucellosis in animals is endemic in most areas of the world, and it can become a serious public health problem that results in significant morbidity and economic losses (Boschiroli et al., 2001; Atluri et al., 2011).

*Brucella* virulence relies mainly on its ability to invade and replicate within professional and non-professional phagocytes, among which macrophages are major target cells in infected mammals (Gorvel and Moreno, 2002; Celli, 2006). To date, many virulence factors have been identified, such as lipopolysaccharide (LPS), the type IV secretion system (T4SS), a two-component regulatory system (BvrS/BvrR), and cyclic β-1,2-glucan (CβG) (Byndloss and Tsolis, 2016). Two major *Brucella* virulence factors are LPS and T4SS. *Brucella* LPS is composed of lipid A, a core oligosaccharide, and the O-antigen. It is characterized by low stimulatory activity and toxicity to cells, and mediates lower superoxide and lysozyme production in infected cells (Goldstein et al., 1992; Rasool et al., 1992). Furthermore, *Brucella* LPS is critical in the inhibition of programmed cell death (apoptosis) and enhances the bacterium′s ability to survive within macrophages (Fernandez-Prada et al., 2003). Rough mutants of *Brucella abortus* that lack the O-antigen, induce infected macrophage death, and are taken up in greater numbers by macrophages than the smooth wild-type strains (Pei and Ficht, 2003; Bronner et al., 2013; Tian et al., 2014). In previous reports, the rough mutant VTRS1 of *B. suis* induced proinflammatory, caspase-2- and nuclear factor kappa B (NF-κB)- mediated macrophage cell death (Chen et al., 2011). Bronner and colleagues subsequently reported that the rough mutant of *B. abortus* RB51 induces a hybrid cell death, mediated by caspase-2 activation, with features of apoptosis and pyroptosis (Bronner et al., 2013). In further study, Bronner found that endoplasmic reticulum (ER) stress induced by RB51 activates the inflammasome via NLRP3- and caspase-2- driven mitochondrial damage (Chen et al., 2011; Bronner et al., 2015). However, the molecular mechanism underlying *Brucella* rough mutant modulation of ER stress to induce macrophage death remains unclear.

Cytotoxicity in macrophages that have been infected by *Brucella* rough mutants is reportedly T4SS dependent (Pei et al., 2008). Overexpression of T4SS in the *Brucella* smooth strain enhances its ability to induce macrophage death (Zhong et al., 2009). The T4SS encoded by the *virB* operon in *Brucella* comprises multiprotein complexes that translocate specific protein substrates across the bacterial cell envelope to the host cell, and guides trafficking of the *Brucella* containing vacuole to the ER-associated compartment within macrophages (Zechner et al., 2012; Byndloss et al., 2016). The *Brucella virB* operon is induced by lysosomal acidification and nutritional deprivation within macrophages, which is tightly controlled by several regulation-associated genes, such as the LuxR family VjbR regulator and integration host factor IHF (Porte et al., 1999; Boschiroli et al., 2002; Sieira, 2013). The T4SS plays a significant role in *Brucella* trafficking, and is essential for *Brucella* to trigger a mild inflammatory response (Rolan et al., 2009). The effector protein VceC is translocated by T4SS to the ER, where it binds the ER chaperone BiP (binding immunoglobulin protein) and induces inositol-requiring enzyme 1α (IRE1α)-dependent ER stress during *B. abortus* infection, and this in turn effects the recruitment of the NOD-like receptors, NOD1 and NOD2, to induce NF-κB activation and expression of proinflammatory genes (de Jong et al., 2013; Keestra-Gounder et al., 2016). However, the manner in which the *Brucella* rough mutant regulates T4SS function has not been definitively determined.

*Brucella* LPS is an important virulence factor, and the O-antigen is a crucial component that is synthesized in the cytoplasmic face of the bacterial inner membrane, and then exported to the periplasmic face of the inner membrane, based on an ATP-binding cassette (ABC) transporter system that is encoded by *rfbE* and *rfbD* genes (Godfroid et al., 2000; Tian et al., 2014). In a previous study, we found that the *B. abortus* rough mutant Δ*rfbE*, induced the death of infected macrophages (Tian et al., 2014), which is in line with the findings of other reports (Pei and Ficht, 2003; Chen et al., 2011). In *Shigella*, shortening of the LPS molecule by O-antigen glucosylation enhances the secretion of effector proteins and function of the type III secretion system (West et al., 2005). In view of these findings, we hypothesized that *Brucella* smooth LPS is crucial for T4SS function, which plays a vital role in intracellular survival of *Brucella* and its interaction with host cells. In this study, we demonstrated that VjbR upregulation in the *Brucella* rough mutant Δ*rfbE* enhances T4SS expression and secretion, both of which contribute to the death of infected macrophages via activation of the IRE1α pathway of ER stress.

## Materials and methods

### Strains, plasmids, macrophages, and culture conditions

All strains and plasmids used in this study are listed in Table 1. *Brucella abortus* S2308 and its derivatives were grown in tryptic soy broth (TSB) or on tryptic soy agar (TSA) (Difco, Franklin Lakes, NJ, USA) plates at 37°C with 5% CO_2_. Manipulation of *Brucella* was performed in a biosafety level 3 laboratory facility at the Chinese Academy of Agricultural Sciences. *Escherichia coli* strains were cultured at 37°C in Luria Broth. When appropriate, 100 μg/mL ampicillin or 20 μg/mL chloramphenicol (Sigma–Aldrich Inc., St. Louis, MO, USA) was added. Mouse macrophage RAW264.7 (ATCC, Manassas, VA, USA) was cultured at 37°C with 5% CO_2_, in Dulbecco's modified Eagle's medium (DMEM) supplemented with 10% heat-inactivated fetal bovine serum (Gibco, ThermoScientific, Grand Island, NY, USA).

**Table 1:** Strains and plasmids used in the present study.

**Strain or plasmid**	**Characteristics**	**Source or references**
**STRAINS**		
*B. abortus* strains		
S2308	wild-type strain; smooth phenotype	ATCC
Δ*rfbE*	*rfbE* deletion mutant strain; rough phenotype	Zhang et al., 2013
Δ*rfbE*(pBBR-*rfbE*)	Δ*rfbE* strain carrying the complementary plasmid pBBR-*rfbE*; smooth phenotype	Tian et al., 2014
Δ*rfbE*Δ*virB*	*rfbE* and *virB123* deletion mutant strain; rough phenotype	This study
Δ*rfbE*Δ*vjbR*	*rfbE* and *vjbR* deletion mutant strain; rough phenotype	This study
S2308(BPE123-Luc)	S2308 based luciferase reporter strain of T4SS effector BPE123; smooth phenotype	This study
Δ*rfbE* (BPE123-Luc)	Δ*rfbE* based luciferase reporter strain of T4SS effector BPE123; rough phenotype	This study
Δ*rfbE*Δ*virB* (BPE123-Luc)	Δ*rfbE*Δ*virB* based luciferase reporter strain of T4SS effector BPE123; rough phenotype	This study
S2308(Luc-VceC)	S2308 based luciferase reporter strain of T4SS effector VceC; smooth phenotype	This study
Δ*rfbE*(Luc-VceC)	Δ*rfbE* based luciferase reporter strain of T4SS effector VceC; rough phenotype	This study
Δ*rfbE*Δ*virB* (Luc-VceC)	Δ*rfbE*Δ*virB* based luciferase reporter strain of T4SS effector VceC; rough phenotype	This study
S2308(GST-Luc)	S2308 based luciferase reporter strain of control protein GST; smooth phenotype	This study
Δ*rfbE*(GST-Luc)	Δ*rfbE* based luciferase reporter strain of control protein GST; rough phenotype	This study
Δ*rfbE*Δ*virB* (GST-Luc)	Δ*rfbE*Δ*virB* based luciferase reporter strain of control protein GST; rough phenotype	This study
S2308(pVirB-Luc)	S2308 based luciferase reporter strain of *virB* promoter; smooth phenotype	This study
Δ*rfbE*(pVirB-Luc)	Δ*rfbE* based luciferase reporter strain of *virB* promoter; rough phenotype	This study
Δ*rfbE* (pMdrA)	Δ*rfbE* based *MdrA* overexpression strain; rough phenotype	This study
Δ*rfbE* (pBlxR)	Δ*rfbE* based *BlxR* overexpression strain; rough phenotype	This study
*E. coli* strain		
DH5α	F^−^φ80*lac*ZΔM15Δ(*lac*ZYA-*arg*F)U169 *rec*A1 *end*A1 *hsd*R17(${\text{r}}_{\text{k}}^{-}$,${\text{m}}_{\text{k}}^{+}$) *pho*A *sup*E44 *thi*-1 *gyr*A96 *rel*A1 λ^−^	Invitrogen
**PLASMIDS**		
pBBR1MCS1	Cm^r^; Broad-host-range cloning vector; parental plasmid	Kovach et al., 1994
pBAD24	Amp^r^; Bacterial expression plasmid	Guzman et al., 1995
pMCR	Cm^r^; The lacZ promoter region was replaced by *rrnB* terminator	This study
pBCSP31	Cm^r^; The promoter of *bcsp31* gene was inserted into the pBBR1-MCS plasmid	This study
pBCSP31-Luc-N	Cm^r^; Target protein was fuse-expressed to firefly luciferase gene in N-terminal	This study
pBCSP31-Luc-C	Cm^r^; Target protein was fuse-expressed to firefly luciferase gene in C-terminal	This study
pVirB	Cm^r^; The promoter of *virB* operon was inserted into the pBBR1-MCS plasmid	This study
pVirB-Luc	Cm^r^; The luciferase gene was inserted into the pVirB plasmid to report the promoter activity of *virB* operon	This study
pBCSP31-BPE123-Luc	Cm^r^; The *bpe123* gene was inserted into the pBCSP31-Luc-N plasmid	This study
pBCSP31-Luc-VceC	Cm^r^; The *vceC* gene of C-terminal 116 amino acids was inserted into the pBCSP31-Luc-C plasmid	This study
pBCSP31-GST-Luc	Cm^r^; The *gst* gene was inserted into the pBCSP31-Luc-N plasmid	This study
pSC	Amp^r^; pUC19 plasmid containing *sacB* gene	
pSCΔ*virB123*	Amp^r^; pSC plasmid containing the *virB123* fragment; used to construct deletion strain	This study
pSCΔ*vjbR*	Amp^r^; pSC plasmid containing the *vjbR* fragment; used to construct deletion strain	This study
pMdrA	Cm^r^; pBBR1 plasmid containing the *MdrA* gene; used to construct overexpression strain	This study
pBlxR	Cm^r^; pBBR1 plasmid containing the *BlxR* gene; used to construct overexpression strain	This study

### Antibodies

The primary antibodies used in this study were: rabbit anti-firefly luciferase monoclonal antibody (Abcam, Cambridge, MA, USA); rabbit anti-*Brucella* VirB5 polyclonal antibody (prepared in our lab); rabbit anti-*Brucella* GAPDH polyclonal antibody (prepared in our lab); rabbit anti-IRE1 polyclonal antibody (phospho S724, Abcam); and rabbit anti-β-actin monoclonal antibody (Cell Signaling Technology, Danvers, MA, USA). The secondary antibodies used for western blotting were: IRDye 800CW-conjugated donkey anti-Rabbit IgG polyclonal antibody (LI-COR Biosciences, Lincoln, NE, USA) and horseradish peroxidase -conjugated goat anti-rabbit IgG (Life Technologies, Eugene, OR, USA).

### Plasmid construction

All primers used in this study are listed in Table 2. Suicide plasmids were constructed, using an overlap PCR assay, as previously reported (Tian et al., 2014). Briefly, the upstream and downstream fragments of *virB123* (containing the *virB* promoter, *virB1, virB2*, and *virB3* genes) and *vjbR* were amplified by independent PCRs and extracted from agarose gels that were used as templates for a second round of PCR. The resultant product that contained joined flanking sequences was purified by gel extraction and cloned into a pSC plasmid, after being digested with XbaI, to generate the suicide plasmids pSCΔ*virB123* and pSCΔ*vjbR*.

**Table 2:** Primers used in the present study.

**Primers**	**Oligonucleotide sequences (5′–3′)^a^**	**Target genes^b^**	**Product size (bp)**
*virB123*-UF	GCTCTAGAGCCATAATCGGAGCCAGCCTTTC	Upstream fragment of *virB123*	1323
*virB123*-UR	AAATCCAGACCGATAAGAGTTACCGAACTTGCTCCATT	Upstream fragment of *virB123*	1323
*virB123*-DF	AATGGAGCAAGTTCGGTAACTCTTATCGGTCTGGATTT	Downstream fragment of *virB123*	1231
*virB123*-DR	GCTCTAGAGCGCAAATACCAGCAGCGAAT	Downstream fragment of *virB123*	1231
*vjbR*-UF	GCTCTAGAGCCCTCCTGCCTGCCTGAAA	Upstream fragment of *vjbR*	895
*vjbR*-UR	GTAGATAAGTCACCACCGTCCCGGGCTTCGCTCTGGTAT	Upstream fragment of *vjbR*	895
*vjbR*-DF	ATACCAGAGCGAAGCCCGGGACGGTGGTGACTTATCTAC	Downstream fragment of *vjbR*	522
*vjbR*-DR	GCTCTAGAGCATTGCCTTCATACGCTGTG	Downstream fragment of *vjbR*	522
*rrnB-*F	GCGCGTTGGCCGATTCATTAAGGCTGTTTTGGCGGATG AGAGAAG	Terminator sequence of *rrnB*	461
*rrnB-*R	GGGGTACCAGAGTTTGTAGAAACGCAAAAAGGC	Terminator sequence of *rrnB*	461
P*_*bcsp*31_*-F	GGGGTACCAAGCGATTGTATTCTTTGG	Fragment of promoter *bcsp31*	112
P*_*bcsp*31_*-R	CCCTCGAGAATACCAGTCCTCTTCCCG	Fragment of promoter *bcsp31*	112
Luc-CF	CGGGATCCATGGAAGATGCCAAAAACAT	Coding region of luciferase	1653
Luc-CR	GCTCTAGATTACACGGCGATCTTGCCGCCCT	Coding region of luciferase	1653
Luc-NF	CCCTCGAGATGGAAGATGCCAAAAACAT	Coding region of luciferase lacking termination codon (TAA)	1650
Luc-NR	TGCACTGCAGCACGGCGATCTTGCCGCCCT	Coding region of luciferase lacking termination codon (TAA)	1650
*BPE123*-F	CCCTCGAGATGAGCTTGTTGCTGGCTAAC	Coding region of *BPE123*	471
*BPE123*-R	CGGGATCCTGCCTGTCCCGCCAGTTCAAC	Coding region of *BPE123*	471
*vceC*-F	TGCACTGCAGCAGCCGGAACGTTCAGAGCG	Coding region of *vceC*	363
*vceC*-R	GCTCTAGATCAATTGCGGGTTTCTCCCT	Coding region of *vceC*	363
*GST*-F	CCCTCGAGATGTCCCCTATACTAGGTTAT	Coding region of *GST*	675
*GST*-R	CGGGATCCACGCGGAACCAGATCCGAT	Coding region of *GST*	675
*mdrA*-F	GGGGTACCATGACCAATACCCAGCGCAAG	Coding region of *mdra*	516
*mdrA*-R	CGGGATCCTTACAGGCGGTAAGCGATGG	Coding region of *mdra*	516
*blxR*-F	GGGGTACC ATGAAATGGGAAACATTTTATG	Coding region of *blxr*	708
*blxR*-R	CGGGATCCTCAGAGGAGACCAAATGTACGG	Coding region of *blxr*	708
P*_*virB*_*-F	CTCTGGTAGGGGTACCATGACAGGCATATTCAACGCGAC	Fragment of promoter *virb*	423
P*_*virB*_*-R	TTGGCATCTTCCATGGATCCCACCATAGGATCGTCTCCTTC	Fragment of promoter *virB*	423
RT-*16S*F	ACGTGCTACAATGGTGGTGA	16SRNA	87
RT-*16S*R	CAGAGTGCAATCCGAACTGA	16SRNA	87
RT-*virB4*F	GCCTGCTCAACTCCAAAGTC	*virB4*	156
RT-*virB4*R	GGCTTTCCTCGCTCATACTG	*virB4*	156
RT-*vjbR*F	CCGCTACGTAACGCATACCT	*vjbR*	172
RT-*vjbR*R	ATTGCGGTAATACGGAGCGT	*vjbR*	172
RT-*ihf* F	TTGCGACATTTCAGGTTCGC	*ihf*	164
RT- *ihf* R	CGAGGTTTTACCCTGACGCT	*ihf*	164
RT-*hutC*F	ACTTGGCCTGCCTTATCGTT	*hutC*	155
RT- *hutC*R	GCTCTTCCAGTGCAAACACG	*hutC*	155
RT- *blxR-*F	TGAAGTGGGTGCGATTTGGA	*blxR*	248
RT- *blxR*R	GCGTACAAAGCTCAAGAGGC	*blxR*	248
RT-*bvrR*F	AGTCGGAAGGTTATCGCGTC	*bvrR*	174
RT-*bvrR*R	AAGTGAGGAAGATGACCGGC	*bvrR*	174
RT-*mdrA*F	GCGACCTGATTGGCGATTTC	*mdrA*	155
RT-*mdrA*R	CGCTCGCGTGGTTACTATCT	*mdrA*	155

a *Underlining indicates restriction endonuclease recognition sequences*.

b *B. abortus locus tags listed are for genes in the B. abortus strain S2308*.

Luciferase reporter plasmids of pBCSP31-BPE123-Luc, pBCSP31-Luc-VceC, pBCSP31-GST-Luc, and pVirB-Luc were constructed using conventional methods. Firstly, the *lacZ* promoter region of the broad-host-range cloning plasmid pBBR1-MCS was replaced by a terminator sequence of *rrnB* from the plasmid pBAD24, using overlap PCR with the primer *rrnB*-F/R. The linearized plasmid was digested by the restriction enzyme Kpn I and self-ligated using T4 DNA ligase to construct a pMCR plasmid. The promoter regions of the *bcsp31* gene or *virB* operon from *B. abortus* strain 2308 were obtained by PCR using the primers P_*bcsp*31_-F/R or P_*virB*_-F/R, cloned into the pMCR plasmid and digested by KpnI and XhoI enzymes to generate the plasmids pBCSP31 and pVirB, respectively. The luciferase gene (*luc*) was amplified by PCR from the pNFκB-Luc plasmid (Beyotime, Jiangsu, China), using the primers Luc-F and Luc-R, and cloned into the XhoI- and PstI-digested pBCSP31 plasmids, to generate pBCSP31-Luc-N, and facilitate the generation of N-terminal in-frame fusions of the Luc protein. The *luc* gene was cloned into the pBCSP31 plasmid by BamHI and XbaI digestion to generate pBCSP31-Luc-C, in which the stop codon in the *luc* open reading frame (ORF) was removed, to allow C-terminal fusion of the Luc protein. Furthermore, the *luc* gene was cloned into the pVirB plasmid by XhoI and PstI digestion, to construct the pVirB-Luc plasmid and effect promoter activation of the *virB* operon. The ORF of BPE123 was amplified from S2308 by PCR using the primer *BPE123*-F/R, and cloned into the pBCSP31-Luc-N plasmid to generate pBCSP31-BPE123-Luc. At the C-terminal, 116 amino acids of the VceC protein that are necessary for secretion by T4SS was amplified by PCR using the primer *vceC*-F/R, and cloned into the pBCSP31-Luc-C plasmid to construct the pBCSP31-Luc-VceC plasmid. In addition, the glutathione transferase (GST) gene was amplified from the pGEX-4T-1 plasmid (Takara, Dalian, China) by PCR using the primer *GST*-F/R and cloned into the pBCSP31-Luc-N plasmid, to generate the negative control plasmid pBCSP31-GST-Luc.

Overexpression plasmids of pMdrA and pBlxR were constructed using conventional methods. The *mdrA* and *blxR* genes containing the promoter and terminator regions were amplified by independent PCRs using the primers *mdrA*-F/R and *blxR*-F/R, respectively, and then cloned into the plasmid pBBR1-MCS, to generate the plasmids pMdrA and pBlxR, respectively.

All recombinant plasmids were propagated in *E. coli* DH5α cells (Invitrogen Corp., Carlsbad, CA, USA) and then extracted to construct recombinant *Brucella* strains.

### Mutant construction

The Δ*rfbE*Δ*virB* and Δ*rfbE*Δ*vjbR* mutants were constructed by allelic replacement, using a two-step strategy as previously reported (Kahl-McDonagh and Ficht, 2006; Tian et al., 2014). The suicide plasmids pSCΔ*virB123* and pSCΔ*vjbR* (0.5–1.0 μg) were transferred to the Δ*rfbE* strain by electroporation. The first exchanged recombinants were selected by plating on TSA containing ampicillin. The second round of exchanged recombinants was selected by plating on TSA containing 5% sucrose. Analyses of PCRs were carried out to identify clones.

Luciferase reporter strains and overexpression strains were also constructed by electroporation. The recombinants were then selected by plating on TSA containing chloramphenicol. The PCR or western blotting analyses were carried out to identify recombinants. The recombinant strains constructed in this study are listed in Table 1.

### Cell infection assay

Monolayers of RAW264.7 cells were cultured in six- or 24-well plates and infected with *B. abortus* S2308 or its derivatives at a multiplicity of infection (MOI) of 100 or 1,000 colony forming units (CFU) per cell. To synchronize the infection, the infected plates were centrifuged at 400× *g* for 5 min, and cells were then incubated at 37°C with 5% CO_2_ for 1 h. The monolayers were washed twice with phosphate buffered saline (PBS) (HyClone, GE Lifesciences, Logan, UT, USA) to remove extracellular nonadherent bacteria, and then incubated with DMEM containing gentamicin (100 μg/mL) for 1 h to kill extracellular bacteria. To maintain survival of the infected cells, the monolayers were incubated with DMEM containing gentamicin (20 μg/mL) and 2% FBS after being washed thrice with PBS.

### Cell death analysis

Macrophage death was detected, using two approaches. In the first approach, infected cells were stained with annexin V and propidium iodide (PI) at 3, 5, 8, and 12 h post infection (p.i.), using the annexin V-FITC/PI staining kit (Beyotime, Shanghai, China). In the second approach, the release of lactate dehydrogenase (LDH) in the supernatant of *Brucella*-infected RAW264.7 cells both with and without 4μ8c (IRE1α inhibitor, 100 μM, Selleck, Houston, TX, USA) treatment was determined at 3, 5, 8, and 12 h p.i., using a CytoTox 96 nonradioactive cytotoxicity assay (Promega, Fitchburg, WI, USA). Cell death was expressed as a percentage of maximum LDH release. The percentage was calculated as follows: (optical density at 490 nm [OD_490_] of infected cells—OD_490_ of uninfected cells)/(OD_490_ of lysed uninfected cells—OD_490_ of uninfected cells) × 100%.

### Determination of luciferase activity

For the determination of luciferase activity in the media culture, luciferase reporter strains of S2308(pVirB-Luc) and Δ*rfbE*(pVirB-Luc) were cultured to exponential phase (OD_600_ = 1.0), and then centrifuged at 8,000 × *g* for 5 min to precipitate bacteria. The pellets of luciferase reporter strains were resuspended in 200 μL PBS and lysed by adding 200 μL B-PER® Bacterial Protein Extraction Reagent (Thermo Scientific). The lysate was centrifuged at 15,000 × *g* for 5 min to separate soluble proteins, after which it was incubated for 15 min at room temperature. The luciferase activity (relative light units, RLUs) of lysate supernatants were measured using the Luc-Screen® reporter gene assay system (Abcam). Moreover, 100 μL of the luciferase reporter strains were serially diluted 10-fold with PBS and spread onto TSA plates to determine the bacterial CFU. All samples were analyzed in triplicate.

For the determination of luciferase activity in the cell culture, macrophage RAW264.7 cells were infected with luciferase reporter strains at a MOI of 1,000, as described previously. Infected cells were washed three times with PBS and lysed with 500 μL of 0.2% Triton X-100 in sterile water for 15 min at 3, 5, and 8 h p.i. The infected cell lysate (400 μL) was centrifuged at 12,000× *g* for 5 min. For cells infected with luciferase reporter strains of S2308(BPE123-Luc), Δ*rfbE*(BPE123-Luc), Δ*rfbE*Δ*virB*(BPE123-Luc), S2308(Luc-VceC), Δ*rfbE*(Luc-VceC), Δ*rfbE*Δ*virB*(Luc-VceC), S2308(GST-Luc), Δ*rfbE*(GST-Luc), and Δ*rfbE*Δ*virB*(GST-Luc), the RLUs of lysate supernatants were measured by the Luc-Screen® reporter gene assay system (Abcam). For the cells infected with luciferase reporter strains of S2308(pVirB-Luc) and Δ*rfbE*(pVirB-Luc), lysate pellets were resuspended in 200 μL PBS, after which 200 μL B-PER® Bacterial Protein Extraction Reagent (Thermo Scientific) was added to lyse the intracellular strains. The lysate of intracellular strains was centrifuged at 15,000× *g* for 5 min, after which it was incubated for 15 min at room temperature. The RLUs of lysate supernatants were also measured using the Luc-Screen® reporter gene assay system (Abcam). Moreover, the remaining 100 μL of infected cell lysates were serially diluted 10-fold with PBS and spread onto TSA plates to determine the bacterial CFU. All samples were analyzed in triplicate.

### RNA extraction and real-time PCR

Total RNA was extracted from bacteria using the TRIzol® RNA Isolation Reagent (Invitrogen) according to the manufacturer's protocol. Genomic DNA contamination was removed through treatment with a Turbo DNA-free kit (Ambion). The RNA quantity and quality were evaluated using the NanoDrop ND-1000 spectrophotometer (NanoDrop Technologies, Inc.). The RNA integrity was assessed by standard denaturing agarose gel electrophoresis, and RNA (1 μg) was reverse transcribed into cDNA, using a PrimeScript RT-PCR kit (Takara) according to the manufacturer's instructions. A 20 μL RT-PCR mixture was made comprising 10 μL 2 × GoTaq qPCR master mix (Promega), 1 μL cDNA, 0.5 μL (each) forward and reverse primers (10 μM each), and 8 μL double-distilled water (ddH_2_O). The mixture was incubated at 95°C for 2 min, and then subjected to 40 cycles at 95°C for 15 s, followed by 60°C for 1 min using a Mastercycler ep Realplex system (Eppendorf). All samples were analyzed in triplicate and relative transcription levels of each gene were determined by the 2^−^ΔΔCt method, using 16S RNA as an internal control for data normalization.

### Western blotting

Sediments of the bacteria were collected, following centrifugation at 1,000 × *g* for 5 min and culture for various durations. The pellets were resuspended in Laemmli sample buffer and boiled for 10 min. The RAW264.7 cells were scraped into radioimmunoprecipitation assay buffer (50 mM Tris, pH 7.4, 150 mM NaCl, 1% NP 40, 0.25% sodium deoxycholate, and 1 mM EDTA) that contained a protease inhibitor cocktail (Roche). The cell lysates were mixed with Laemmli sample buffer and boiled for 10 min. The proteins were separated by sodium dodecyl sulfate-polyacrylamide gel electrophoresis (SDS-PAGE) and then transferred onto nitrocellulose membranes (Millipore) using a semidry transfer procedure. The membranes were blocked overnight at 4°C in Tris-buffered saline containing 5% skim milk or 5% Bovine Serum Albumin (BSA). Immunodetection of proteins in total cell lysates was performed with the respective primary antibody for 2 h at room temperature. After washing three times with Tris-buffered saline and Tween 20, the membrane was incubated with the respective secondary antibody for 1 h at room temperature. After washing three times with Tris-buffered saline and Tween 20, an Odyssey two-color infrared imaging system (LI-COR Biosciences) was used to develop the fluorescence for visualization. The gray intensity of the bands was quantified using the ImageJ software (National Institutes of Health, Bethesda, MD, USA).

### Statistical analysis

Statistical analysis was performed using the GraphPad Prism 6.0 software (GraphPad Software Inc., La Jolla, CA, USA). All *p*-values between identified samples were generated using unpaired two-tailed Student's *t*-tests, or in the case of groups, two-way analysis of variance, followed by the Tukey's test. All experiments were repeated at least three times and the results were presented as means ± SD from ≥3 replicates per condition.

## Results

### Rough mutant Δ*rfbE* induced macrophage death is T4SS dependent

**Figure 1:**
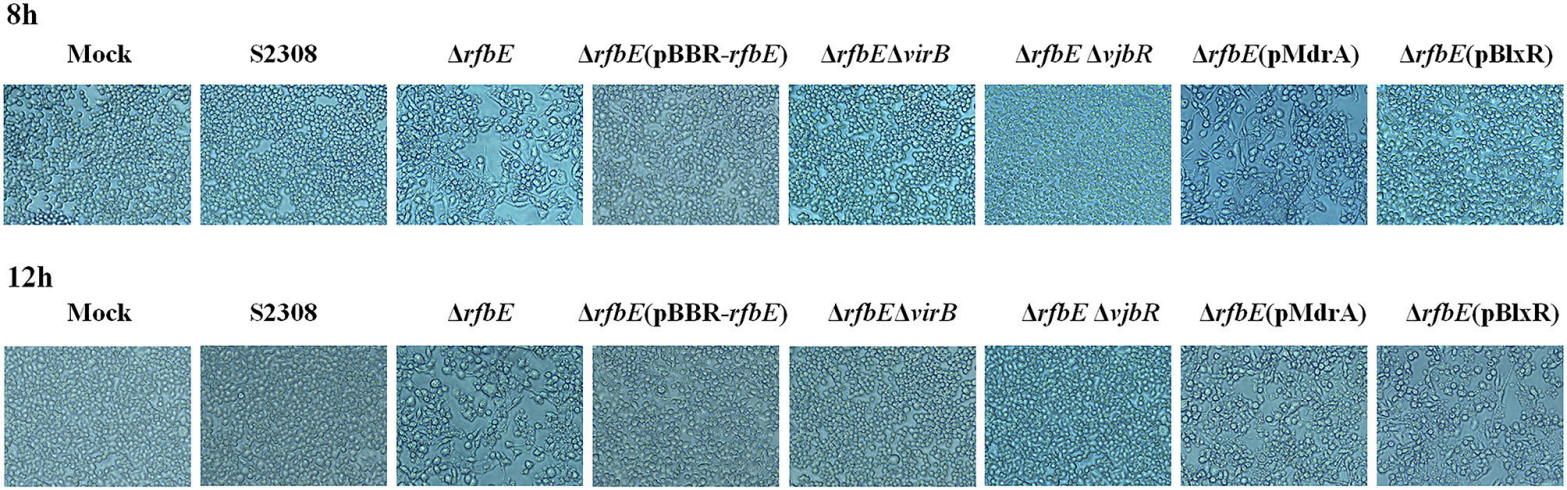
Phase-contrast microscopy. RAW264.7 cells cultured in a 24-well plate were infected with S2308, Δ*rfbE*, Δ*rfbE*(pBBR-*rfbE*), Δ*rfbE*Δ*virB*, Δ*rfbE*Δ*vjbR*, Δ*rfbE*(pMdrA), and Δ*rfbE*(pBlxR) mutants at a MOI of 100. The cells were observed at a magnification of ×200 at 8 and 12 hpi. Uninfected RAW264.7 cells were used as negative controls (Mock).

**Figure 2:**
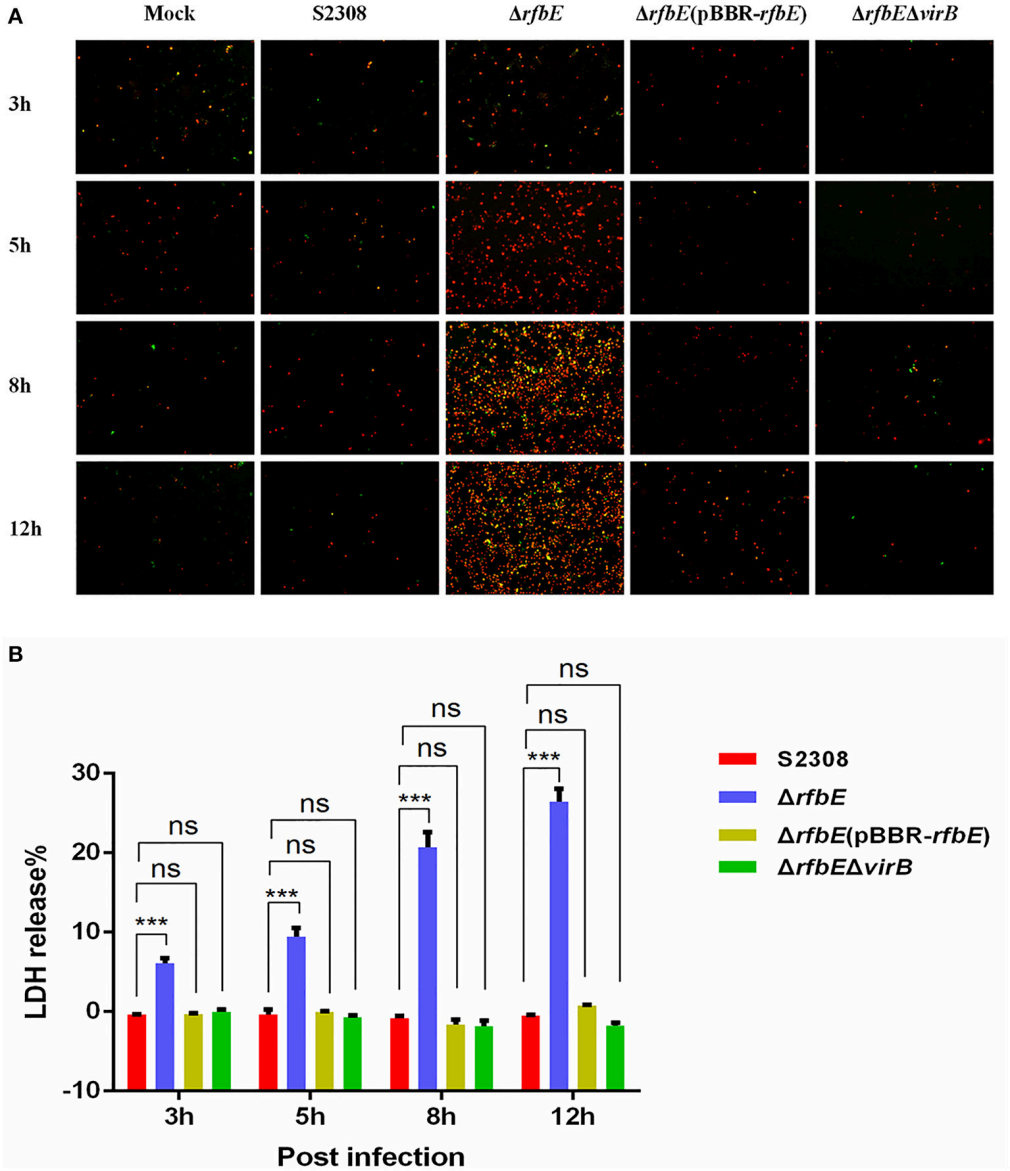
Macrophage death induced by *Brucella* rough mutant infection is T4SS dependent. RAW264.7 cells cultured in a 24-well plate were infected with S2308, Δ*rfbE*, Δ*rfbE*(pBBR-*rfbE*), or Δ*rfbE*Δ*virB* strains at a MOI of 100, and cell death was determined at 3, 5, 8, and 12 hpi. **(A)** Annexin V-FITC/PI staining. The cells were stained with FITC-annexin (green) and PI (red), and observed using fluorescence microscopy at a magnification of ×100. Uninfected RAW264.7 cells were used as negative controls (Mock). **(B)** LDH detection. The supernatants were collected and LDH release was detected using the CytoTox 96 nonradioactive cytotoxicity assay. The supernatants of uninfected RAW264.7 cells were used as negative controls (medium). ns, no significant difference, ^***^p < 0.0001.

*Brucella* rough mutant induces macrophage death, a process that is T4SS dependent (Pei et al., 2008). Our previous studies have shown that rough mutant Δ*rfbE* also induces the death of macrophages (Tian et al., 2014). To identify the role of T4SS on Δ*rfbE* mutant-induced cytotoxicity for RAW264.7 macrophages, *virB1, virB2*, and *virB3* genes were deleted from the Δ*rfbE* mutant, thereby generating a double-knockout strain (Δ*rfbE*Δ*virB*). Firstly, morphology of the RAW264.7 cells infected with S2308, Δ*rfbE*, Δ*rfbE*(pBBR-*rfbE*), and Δ*rfbE*Δ*virB* were observed via light microscopy. As shown in Figure 1, Δ*rfbE-*infected cells exhibited obvious cell swelling and deformation at 8 and 12 h p.i.; however, the Δ*rfbE*(pBBR-*rfbE*)- and Δ*rfbE*Δ*virB-*infected cells showed no cell lesions, which is consistent with our observations of the S2308-infected cells and mock cells. Furthermore, the death of *Brucella*-infected RAW264.7 macrophages was analyzed following annexin V-FITC and PI staining, which was used to detect translocation of phosphatidylserine from the inner cell membrane to the outer cell membrane during the early stages of apoptosis. The PI stains the DNA of necrotic cells and/or cells at the late stage of apoptosis (Tian et al., 2014). The results showed that macrophages infected with the Δ*rfbE* mutant exhibited some characteristics of necrosis and late apoptosis, accompanied by cellular membrane damage and PI staining of the nucleus at 5, 8, and 12 h p.i. Further disruption of the *virB* operon in the Δ*rfbE* mutant reduced its ability to induce cell death. Certainly, the S2308 and Δ*rfbE*(pBBR-*rfbE*) with the smooth phenotype did not induce infected macrophage death in a similar manner to the mock cells (Figure 2A). These results are consistent with those of previous reports (Pei et al., 2008; Tian et al., 2014).

To evaluate cell death quantitatively, the release of LDH was determined for S2308-, Δ*rfbE-*, Δ*rfbE*(pBBR-*rfbE*)-, and Δ*rfbE*Δ*virB*-infected cells. The levels of LDH released from the Δ*rfbE*-infected cells were significantly higher than those released from S2308-infected cells at 3, 5, 8, and 12 h p.i. (Figure 2B). However, the Δ*rfbE*(pBBR-*rfbE*)- and Δ*rfbE*Δ*virB*-infected cells released similar levels of LDH as the S2308-infected cells at 3, 5, 8, and 12 h p.i. (Figure 2B). These results further confirmed that the rough mutant Δ*rfbE* induced macrophage death is T4SS dependent.

### T4SS secretion is enhanced in the *Brucella* rough mutant Δ*rfbE*

**Figure 3:**
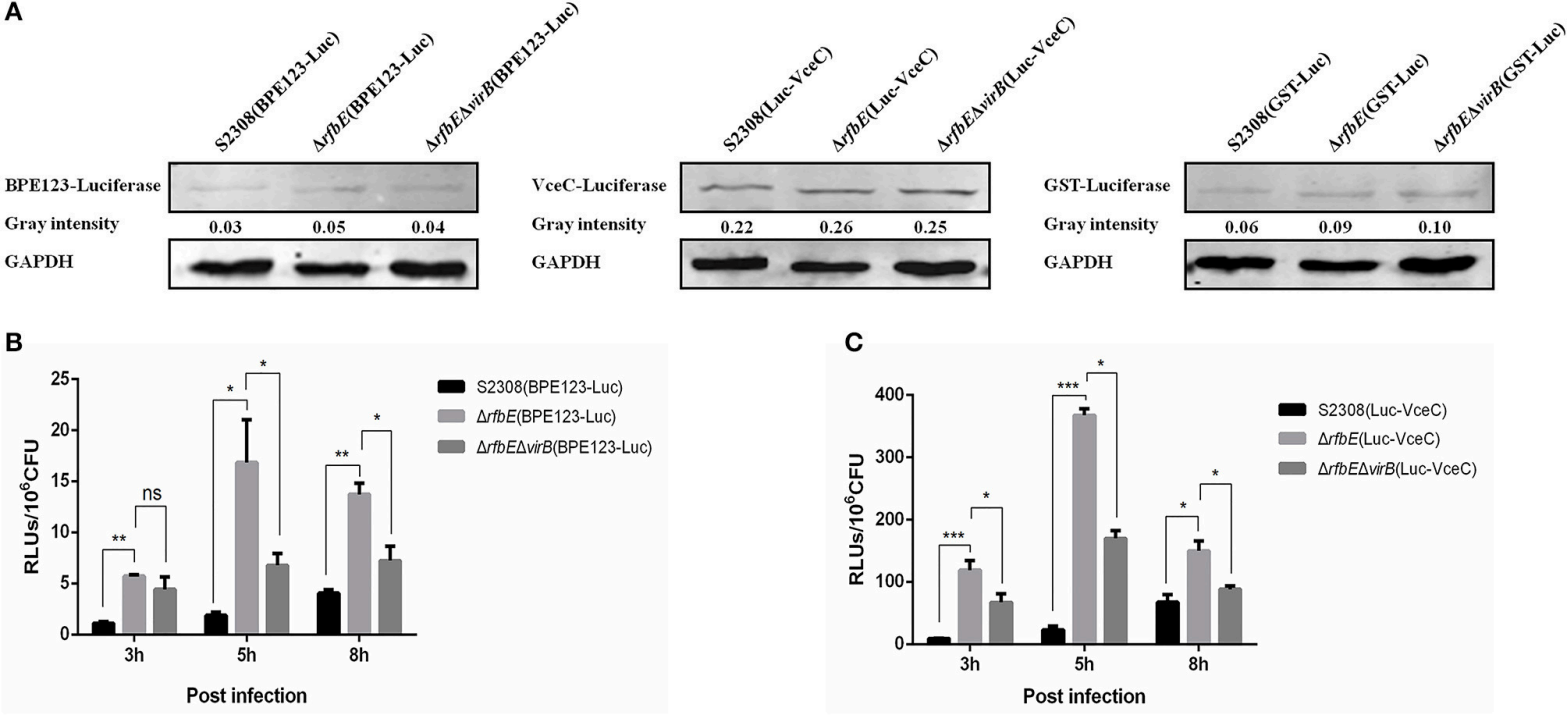
T4SS secretion is enhanced in *Brucella* rough mutant. **(A)** Determination of the T4SS effectors BPE123 and VceC in luciferase reporter strains. No significant difference on BPE123 or VceC expression among S2308(BPE123-Luc), Δ*rfbE*(BPE123-Luc), and Δ*rfbE*Δ*virB*(BPE123-Luc), or S2308(Luc-VceC), Δ*rfbE*(Luc-VceC), and Δ*rfbE*Δ*virB*(Luc-VceC), as determined using western blotting analysis. Protein levels are indicated by gray-scanning intensity values. GAPDH was used for normalization. **(B)** Determination of the BPE123 secretion on cell culture. RAW264.7 cells cultured in a 24-well plate were infected with S2308(BPE123-Luc), Δ*rfbE*(BPE123-Luc), or Δ*rfbE*Δ*virB*(BPE123-Luc) at a MOI of 1,000, and the cells were lysed with 500 μl of 0.2% Triton X-100 in sterile water for 15 min at 3, 5, and 8 hpi. The RLUs of lysate supernatants was measured by the Luc-Screen® reporter gene assay system. ns, no significant difference, ^*^p < 0.05 and ^**^p < 0.001. RAW264.7 cells infected with S2308(GST-Luc), Δ*rfbE*(GST-Luc), and Δ*rfbE*Δ*virB*(GST-Luc) were used as negative controls, respectively. **(C)** Determination of the VceC secretion on cell culture. RAW264.7 cells cultured in a 24-well plate were infected with S2308(Luc-VceC), Δ*rfbE*(Luc-VceC), or Δ*rfbE*Δ*virB*(Luc-VceC) at a MOI of 1000, and the cells were lysed with 500 μl of 0.2% Triton X-100 in sterile water for 15 min at 3, 5, and 8 hpi. The RLUs of lysate supernatants was measured by the Luc-Screen® reporter gene assay system. ^*^p < 0.05 and ^***^p < 0.0001. RAW264.7 cells infected with S2308(GST-Luc), Δ*rfbE*(GST-Luc), and Δ*rfbE*Δ*virB*(GST-Luc) were used as negative controls, respectively.

The T4SS translocates effectors across the bacterial cell envelope to the host cell, and plays a central role in intracellular survival and replication of *Brucella* within the host (Ke et al., 2015). As macrophage death induced by the rough mutant Δ*rfbE* is T4SS dependent, we hypothesized that the capacity of the rough mutant Δ*rfbE*, to secrete T4SS could be altered. For this purpose, we used the previously reported T4SS effector, BPE123 and VceC as target proteins, and GST as a negative control protein, to construct the luciferase reporter strains S2308(BPE123-Luc), Δ*rfbE*(BPE123-Luc), Δ*rfbE*Δ*virB*(BPE123-Luc), S2308(Luc-VceC), Δ*rfbE*(Luc-VceC), Δ*rfbE*Δ*virB*(Luc-VceC), S2308(GST-Luc), Δ*rfbE*(GST-Luc), and Δ*rfbE*Δ*virB*(GST-Luc). The expression of luciferase fusion proteins was detected by western blotting analysis, indicating that luciferase was successfully expressed in the luciferase reporter strains, and the expression levels of luciferase fusion proteins in S2308, Δ*rfbE*, and Δ*rfbE*Δ*virB* were similar (Figure 3A).

Furthermore, the T4SS secretion capacity of S2308, Δ*rfbE*, and Δ*rfbE*Δ*virB* strains within host cells were determined. The RAW264.7 cells were infected with the luciferase reporter strains at a MOI of 1,000, and the secretion of BPE123 and VceC per 10^6^ CFU of intracellular live *Brucella* were determined. Results showed that the rough mutant strain Δ*rfbE* translocated significantly higher levels of BPE123 and VceC to the infected cells than its smooth wild-type strain S2308 at 3, 5, and 8 h p.i., indicating an increased T4SS secretion capacity of the rough mutant Δ*rfbE* under the conditions of intracellular infection (Figures 3B,C). The increased T4SS secretion of the Δ*rfbE* mutant was partially recovered by further deletion of *virB123* genes (Figures 3B,C), indicating that BPE123 and VceC oversecretion in the Δ*rfbE* mutant was indeed dependent on T4SS function. Taken together, the T4SS secretion capacity of the rough mutant, Δ*rfbE* was higher than that of the smooth wild-type strain, S2308.

### T4SS overexpression in the *Brucella* rough mutant contributes to its enhanced secretion

**Figure 4:**
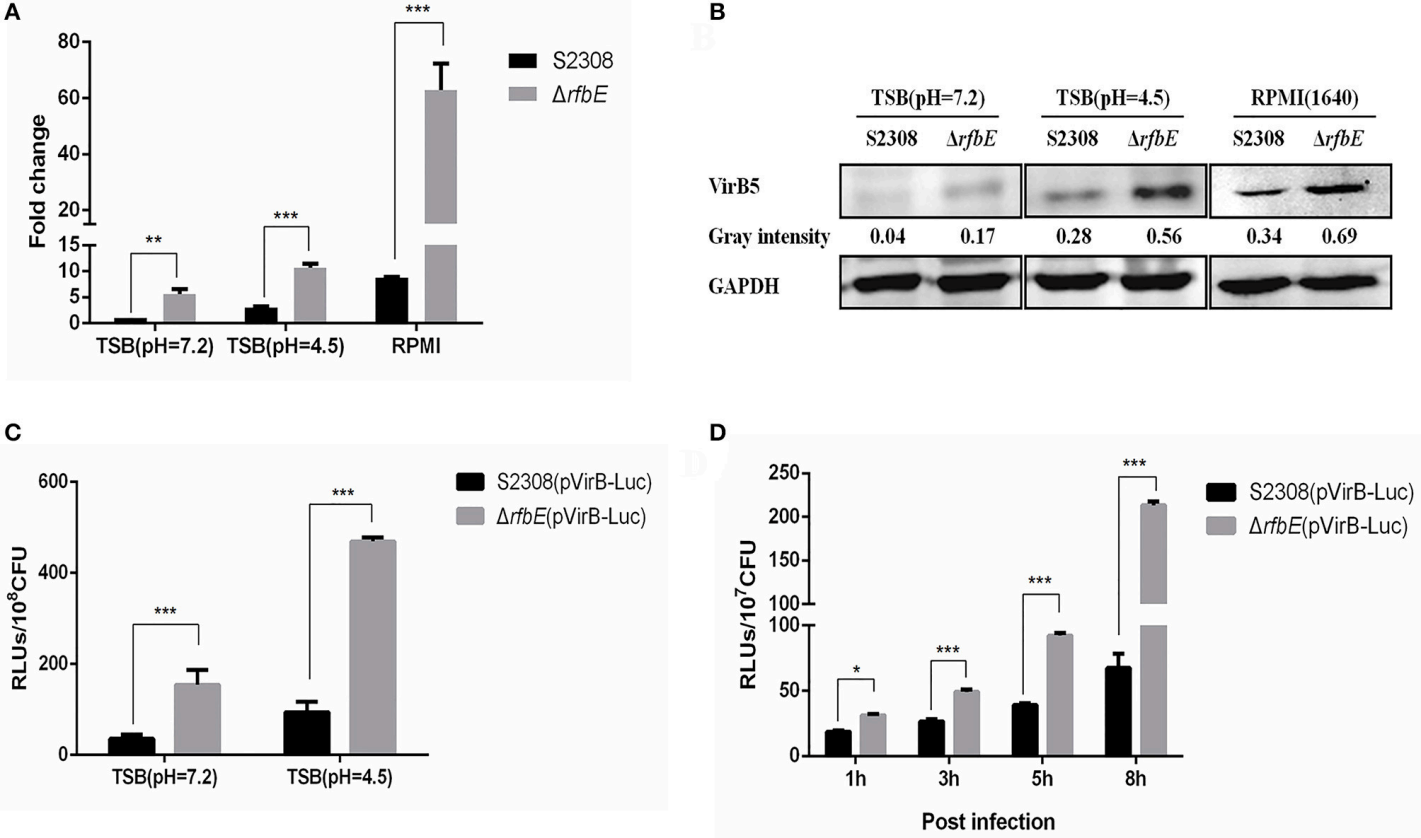
T4SS gene over-expressed in the *Brucella* rough mutant. **(A)** The qRT-PCR analysis. *VirB4* expression in the rough mutant Δ*rfbE* is significantly upregulated under TSB (pH = 7.2), TSB (pH = 4.5), and RPMI 1640, compared to that in the smooth wild-type strain S2308. ^**^p < 0.001 and ^***^p < 0.0001. **(B)** Western blotting analysis. VirB5 protein level in the rough mutant Δ*rfbE* is significantly enhanced under TSB (pH = 7.2), TSB (pH = 4.5), and RPMI 1640, compared to that in the smooth wild-type strain S2308. GAPDH was used for normalization and protein levels are indicated by gray-scanning intensity values. **(C)** Luciferase activity in TSB culture. Luciferase reporter strains S2308(pVirB-Luc) and Δ*rfbE*(pVirB-Luc) were cultured to exponential phase (OD_600_ = 1.0) under vegetative conditions, and the RLUs of culture supernatants was measured by the Luc-Screen® reporter gene assay system. ^***^p < 0.0001. **(D)** Luciferase activity in cell culture. RAW264.7 cells cultured in a 24-well plate were infected with S2308(pVirB-Luc) and Δ*rfbE*(pVirB-Luc) at a multiplicity of infection of 1,000, and the cells were lysed with 500 μl of 0.2% Triton X-100 in sterile water for 15 min at 1, 3, 5, and 8 hpi, and the intracellular bacteria were lysed with B-PER® Bacterial Protein Extraction Reagent. The RLUs of bacterial lysate supernatants was measured by the Luc-Screen® reporter gene assay system. ^*^p < 0.05 and ^***^p < 0.0001.

To confirm that the enhanced T4SS secretion is associated with enhanced T4SS expression in the *Brucella* rough mutant, we evaluated the expression of the T4SS components, VirB4 and VirB5 in the S2308 and Δ*rfbE* mutants at exponential phase in TSB, using qRT-PCR and western blotting. Results showed that *virB4* and *virB5* expression of the rough mutant Δ*rfbE* was significantly upregulated at the exponential phase, compared to that of its smooth wild-type strain, S2308 (Figures 4A,B). Based on previous reports, it is evident that the *virB* operon of *Brucella* induced expression within host cells under the conditions of nutritional deprivation and an acidic environment (Boschiroli et al., 2002). To determine the T4SS expression under acidic conditions, the smooth wild-type strain S2308 and rough mutant Δ*rfbE* were grown to exponential phase and exposed to TSB at pH 4.5 for 1 h. The qRT-PCR and western blotting analyses showed that *virB4* and *virB5* expression was induced in both strains at pH 4.5. Furthermore, much higher levels of *virB4* and *virB5* expression were induced in the rough mutant Δ*rfbE*, compared to those of the smooth wild-type strain S2308 (Figures 4A,B). To determine T4SS expression in nutritional deprivation, the S2308 and rough mutant Δ*rfbE* were grown to log phase and exposed to RPMI 1640 for 3 h. The results showed that *virB4* and *virB5* expression was also induced in both strains, and the expression level of the Δ*rfbE* mutant was significantly higher than that of the S2308 mutant (Figures 4A,B).

To further determine T4SS upregulation in the Δ*rfbE* mutant, the promoter region of the *virB* operon was cloned and fused to the reporter *luc* gene, to generate luciferase reporter strains S2308(pVirB-Luc) and Δ*rfbE*(pVirB-Luc). The promoter activity of the *virB* operon was assessed in both strains during the exponential phase in TSB, indicating that the Δ*rfbE*(pVirB-Luc) strain displayed higher levels of luciferase activity than the S2308(pVirB-Luc) strain (Figure 4C). Furthermore, when exposed to TSB at pH 4.5 for 1 h, luciferase activity in both strains was significantly increased; however, the Δ*rfbE*(pVirB-Luc) strain showed much higher levels of luciferase activity than the S2308(pVirB-Luc) strain (b). To compare promoter activity of both stains within host cells, RAW264.7 cells were infected with S2308(pVirB-Luc) and Δ*rfbE*(pVirB-Luc), at 1, 3, 5, and 8 h p.i. The Δ*rfbE*(pVirB-Luc) strain showed significantly enhanced levels of luciferase activity in comparison to the S2308(pVirB-Luc) strain (Figure 4D). All these data suggest that T4SS expression in the rough mutant Δ*rfbE* was upregulated, which may have contributed to enhanced T4SS secretion.

### Up-regulation of T4SS promoted by VjbR in rough mutant Δ*rfbE* contribute to macrophage death

**Figure 5:**
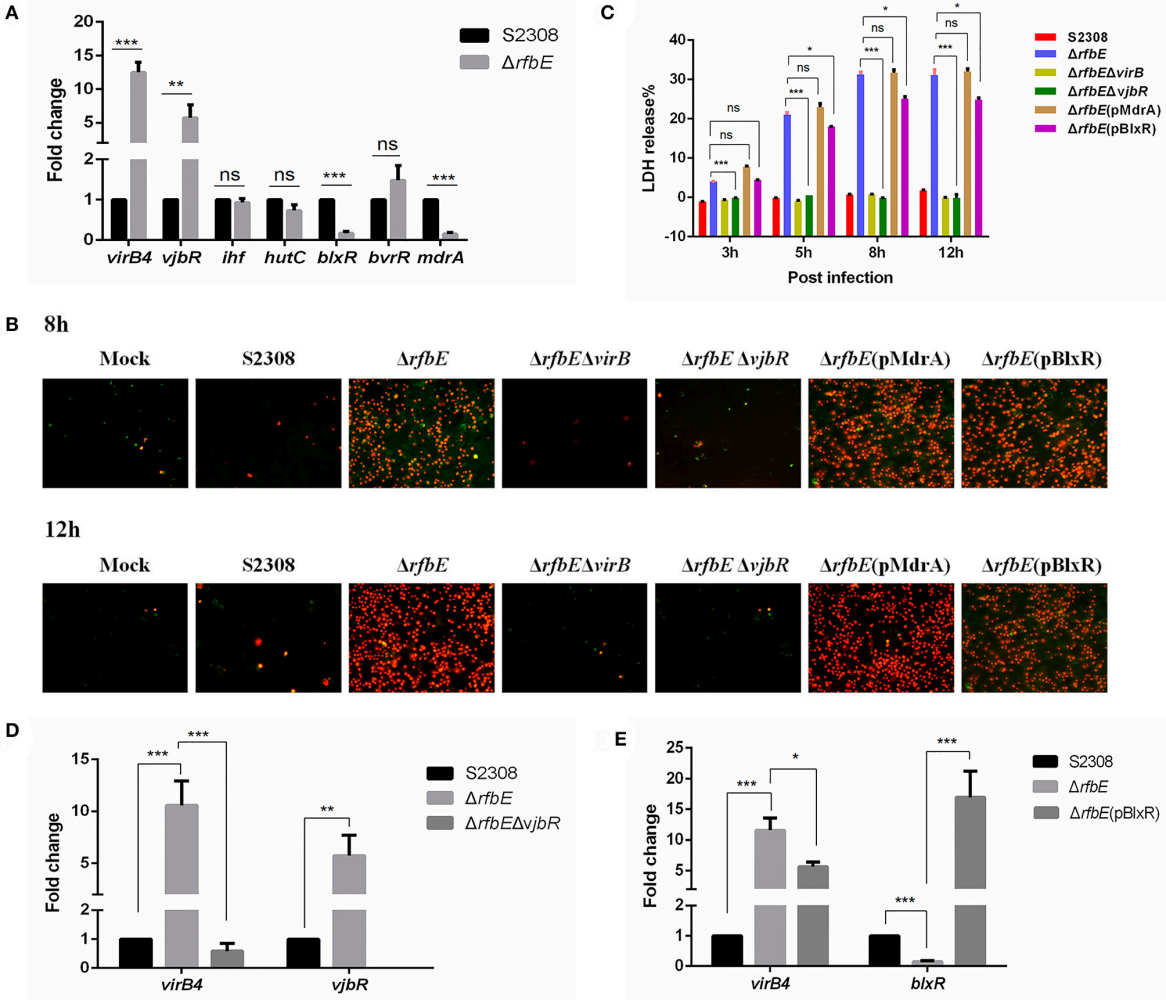
*Brucella* VjbR regulates T4SS expression in the rough mutant to induce macrophage death. **(A)** The qPCR analysis. Compared to the smooth wild-type strain S2308, upregulation of *vjbR* and downregulation of *mdrA* and *blxR* were evident in the rough mutant Δ*rfbE*. ns, no significant difference, ^**^p < 0.001 and ^***^p < 0.0001. **(B)** Annexin V-FITC/PI staining. RAW264.7 cells cultured in a 24-well plate were infected with S2308, Δ*rfbE*, Δ*rfbE*Δ*virB*, Δ*rfbE*Δ*vjbR*, Δ*rfbE*(pMdrA), or Δ*rfbE*(pBlxR) at a multiplicity of infection (MOI) of 100. The cells were stained at 8 and 12 hpi with FITC-annexin (green) and PI (red) and observed by fluorescence microscopy at a magnification of ×200. Uninfected RAW264.7 cells were used as negative controls (Mock). **(C)** Determination of LDH release. RAW264.7 cells were infected with S2308, Δ*rfbE*, Δ*rfbE*Δ*virB*, Δ*rfbE*Δ*vjbR*, Δ*rfbE*(pMdrA), and Δ*rfbE*(pBlxR) at an MOI of 100. The supernatants were collected at 8 and 12 hpi, and LDH release was detected using the CytoTox 96 nonradioactive cytotoxicity assay. The supernatants of uninfected RAW264.7 cells were used as negative controls (medium). ns, no significant difference, ^*^p < 0.05 and ^***^p < 0.0001. **(D)** *VirB* expression in the Δ*rfbE*Δ*vjbR* was recovered to the similar level of the smooth wild-type strain S2308, as determined by qRT-PCR. ^**^p < 0.001 and ^***^p < 0.0001. **(E)** *VirB* expression in the Δ*rfbE*(pBlxR) was partly recovered, compared to the Δ*rfbE* mutant, as determined by qRT-PCR. ^*^p < 0.05 and ^***^p < 0.0001.

We proved that the *virB* operon was upregulated at the transcriptional level in the rough mutant, and further investigated whether T4SS overexpression in the rough mutant is associated with transcriptional regulators that directly bind to the *virB* operon. Thus, we evaluated the transcriptional expression of *Brucella* regulatory proteins that were found to be directly involved in transcriptional regulation of *virB* expression, including VjbR, IHF, HutC, BlxR, BvrR, and MdrA (Sieira, 2013). The qRT-PCR demonstrated that expression of *vjbR* was significantly upregulated, and that of the *mdrA* and *blxR* were evidently downregulated in the Δ*rfbE* mutant, compared to the S2308 strain (Figure 5A).

To determine whether macrophage death caused by infection with the Δ*rfbE* mutant is associated with VjbR, MdrA, and BlxR, we constructed Δ*rfbE*Δ*vjbR*, Δ*rfbE*(pMdrA), and Δ*rfbE*(pBlxR) strains, respectively, to infect RAW264.7 macrophages. Under light microscopy, we observed no morphological changes in Δ*rfbE*Δ*vjbR*-infected macrophages; however, obvious cell swelling and deformation were observed in Δ*rfbE*(pMdrA)- and Δ*rfbE*(pBlxR)-infected cells at 8 and 12 h p.i. (Figure 1). Furthermore, cell death was analyzed following annexin V-FITC and PI staining. The results showed that the Δ*rfbE*Δ*vjbR* mutant was no longer cytotoxic to macrophages; however, the Δ*rfbE*(pMdrA) and Δ*rfbE*(p*BlxR*) strains induced macrophage death at 8 and 12 h p.i. (Figure 5B). In addition, the LDH release assay was performed to assess quantitatively the death of macrophages infected with the Δ*rfbE*Δ*vjbR*, Δ*rfbE*(pMdrA), and Δ*rfbE*(pBlxR) strains. The results showed that the Δ*rfbE*Δ*vjbR* mutant-infected macrophages reduced LDH release, compared to the Δ*rfbE* mutant infected cells, but similar to the smooth wild-type strain S2308 infected cells at 3, 5, 8, and 12 h p.i. Furthermore, the Δ*rfbE*(pBlxR)-infected cells also reduced LDH release at 5, 8, and 12 h p.i. compared to the Δ*rfbE* mutant; however, the LDH levels were much higher than those released from the S2308 infected cells (Figure 5C). The LDH release from Δ*rfbE*(pMdrA) infected cells showed no difference with those from the Δ*rfbE* mutant infected cells (Figure 5C). Taken together, these results indicated that VjbR upregulation was the key cause of Δ*rfbE* mutant-induced macrophage death. The BlxR downregulation played a partial role in macrophage death, but MdrA downregulation was not necessary for the Δ*rfbE* mutant to induce macrophage death.

To determine whether *vjbR* and *blxR* are essential for *virB*-upregulated expression in the Δ*rfbE* mutant, Δ*rfbE*Δ*vjbR*, and Δ*rfbE*(pBlxR) strains were evaluated for *virB* expression using qRT-PCR. Results demonstrated that deletion of *vjbR* in the Δ*rfbE* mutant restored *virB4* transcription to a level similar to that of the smooth strain S2308 (Figure 5D). In comparison to the Δ*rfbE* mutant, *virB4* was significantly downregulated when *blxR* was robustly over-expressed in the Δ*rfbE*(pBlxR) mutant (Figure 5E), suggesting that *virB*-upregulated expression in the Δ*rfbE* mutant was associated with the regulatory proteins, VjbR and BlxR.

Taken together, T4SS overexpression induced by VjbR regulation in the *Brucella* rough mutant plays a key role in macrophage death.

### Rough mutant Δ*rfbE* induces macrophage death via activating IRE1α pathway of ER stress

**Figure 6:**
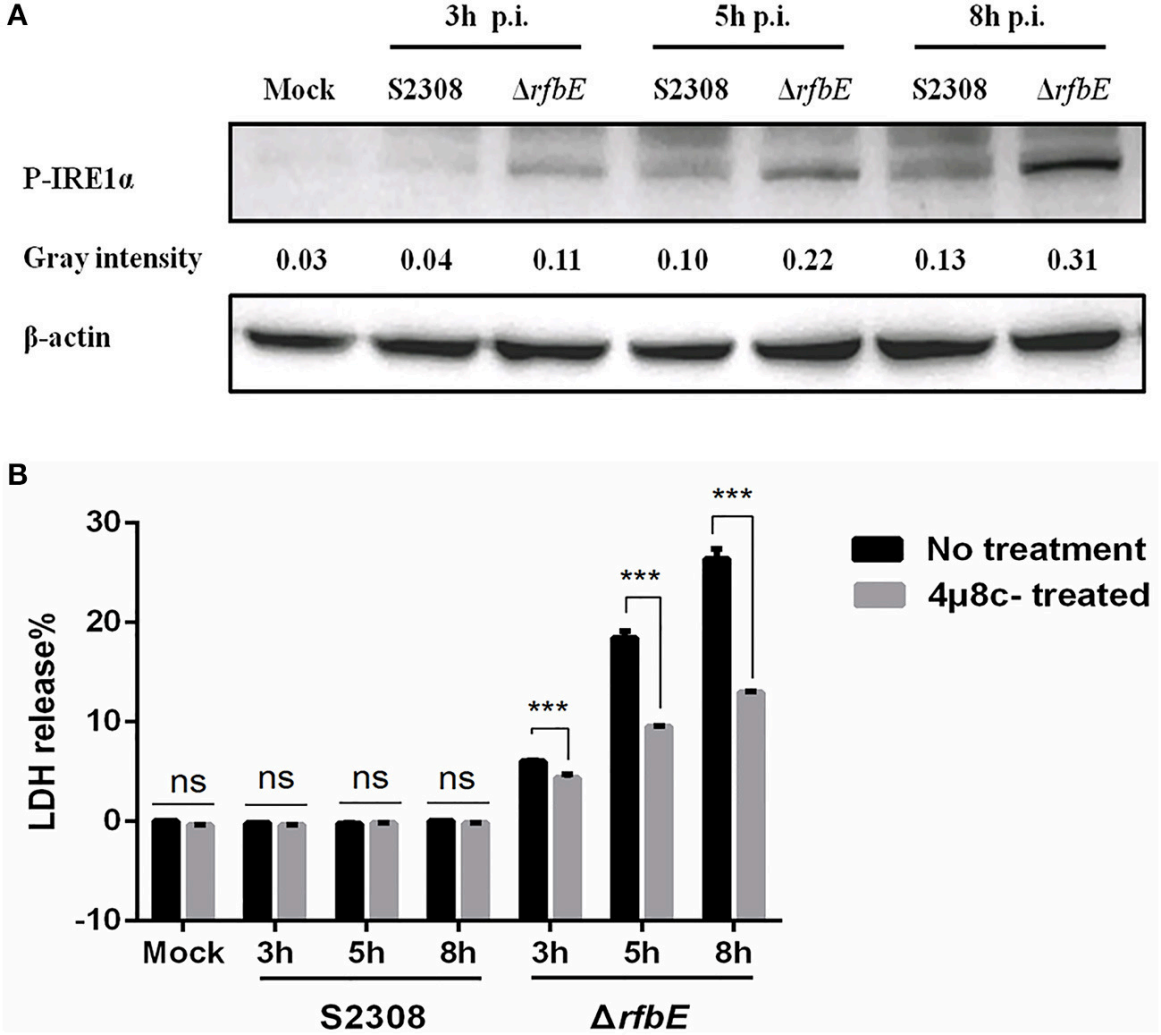
The *Brucella* rough mutant induces stronger ER stress, which plays a role in macrophage death. **(A)** Western blotting analysis. RAW264.7 cells were infected with S2308 or Δ*rfbE* at a MOI of 100. Cell lysates were collected at 3, 5, and 8 hpi, and representative immunoblots for P-IRE1α were analyzed. β-actin was used for normalization. Uninfected RAW264.7 cells were used as negative controls (Mock). The intensity of the bands was quantified using the ImageJ software. **(B)** Determination of LDH release. RAW264.7 cells both with and without 4μ8c (IRE1α inhibitor, 100 μM) treatment were infected with either S2308 or the Δ*rfbE* mutant at an MOI of 100. The supernatants were collected at 3, 5, and 8 hpi, and LDH release was detected using the CytoTox 96 nonradioactive cytotoxicity assay. The supernatants of uninfected RAW264.7 cells were used as negative controls (medium). ns, no significant difference, ^***^p < 0.0001.

The *Brucella* T4SS effector protein, VceC, is associated with triggering ER stress by activating the unfolded protein response (UPR) sensor, inositol-requiring enzyme 1α (IRE1α) (de Jong et al., 2013; Keestra-Gounder et al., 2016). To investigate the ER stress induced by *Brucella* rough mutant infection, activation of the UPR sensor, IRE1α was analyzed using western blotting. The results showed that compared to the *Brucella* smooth wild-type strain, the levels of P-IRE1α in the *Brucella* rough mutant were significantly increased at 3, 5, and 8 h p.i. (Figure 6A), indicating that the *Brucella* rough mutant induced stronger ER stress. To determine whether P-IRE1α is involved in macrophage death caused by the Δ*rfbE* mutant, the inhibitor of IRE1α, 4μ8c, was used to treat the macrophages before infection, which blocks the access of the substrate to the active site of IRE1α, and selectively inactivates both Xbp1 splicing and IRE1α-mediated mRNA degradation (Cross et al., 2012). We infected 4μ8c-treated macrophages with S2308 and the Δ*rfbE* mutant, and then evaluated cell death quantitatively, using the LDH release assay. The results demonstrated that in comparison to macrophages that had not been subjected to 4μ8c treatment, LDH levels were diminished in 4μ8c-treated macrophages infected with the Δ*rfbE* mutant at 3, 5, and 8 h p.i. (Figure 6B), indicating that IRE1α inhibition reduced macrophage death caused by the Δ*rfbE* mutant. The 4μ8c treatment did not affect LDH release from the macrophages infected with S2308 at 3, 5, and 8 h p.i. (Figure 6B).

## Discussion

The cytotoxicity induced by *Brucella* rough mutants within macrophages was originally described more than 50 years ago (Freeman et al., 1961; Freeman and Rumack, 1964). The T4SS is essential for cytotoxic death of macrophages induced by *Brucella* infection (Pei et al., 2008). *Brucella* T4SS is tightly regulated by various regulatory proteins under specific conditions, such as acidification and nutritional deprivation. Deletion or overexpression of *virB* is detrimental to intracellular survival of *Brucella* (Zhong et al., 2009). In addition, shortening of the LPS molecule enhances the type III secretion system in *Shigella* (West et al., 2005). In this study, we confirmed that the capacity for T4SS secretion and the effectors being translocated to macrophages were highly increased in the Δ*rfbE* mutant.

The *virB* mRNA level has been shown to be very low when *Brucella* is grown in a rich medium at neutral pH, and *virB* transcription is upregulated when cultured in acidic conditions or minimal medium (Boschiroli et al., 2002). However, the use of *lacZ* reporter gene fusions has shown that the *virB* operon of *B. abortus* S2308 is expressed during the stationary phase, without the requirement for acidic induction conditions (Sieira et al., 2000). According to the analysis of mRNA levels, our results demonstrated that in comparison to the smooth wild-type strain S2308, T4SS expression of the rough mutant Δ*rfbE* was significantly upregulated at the exponential phase under conditions of both a rich medium at neutral pH and nutrient-deprived or acidic conditions. In the rich medium at neutral pH, the parental *B. abortus* and *B. melitensis* strains constitutively produced *virB5* and *virB8* (Rouot et al., 2003). However, in this study, the smooth wild-type strain, S2308, produced a low level of the *virB5* protein in a rich medium at neutral pH; whereas *virB5* expression levels of the rough mutant, Δ*rfbE*, were significantly increased. After exposure to acidic minimal medium, *Brucella* easily produces detectable levels of *virB8* (Rouot et al., 2003). Under nutrient-deprived or acidic conditions, we confirmed that the *virB5* protein is easily detected, and that expression of *virB5* in the rough mutant Δ*rfbE*, was higher than that in the S2308 strain. Thus, enhanced expression of T4SS in the Δ*rfbE* mutant might account for its increased capacity for T4SS secretion.

Intracellular induction of *virB* expression has been observed to be transient, and the translocation and activity of VirB-secreted effectors within the host cell might be determined by the timing of expression of the *virB* operon (Sieira, 2013). In this work, we analyzed the activity of the *virB* promoter in the smooth wild-type strain S2308, and its rough mutant Δ*rfbE*, using the luciferase reporter assay in a rich medium and in an intracellular environment. In compared to the wild-type strain S2308, the *virB* promoter activity of the rough mutant Δ*rfbE*, was significantly increased in the rich medium and in acidic conditions, both of which enhanced T4SS expression and secretion in the rough mutant Δ*rfbE*. Once *Brucella* is internalized in macrophages, the transcriptional activity of the *virB* promoter reaches a maximum level at 5 h p.i., and the promoter is then turned off, when *Brucella* reaches its replicative niche (Sieira et al., 2004). Our results demonstrated that the activity of the *virB* promoter was increased in the intracellular environment of both the smooth wild-type strain S2308, and the rough mutant Δ*rfbE*, at an early stage of infection. Furthermore, T4SS expression and secretion of the Δ*rfbE* mutant was notably upregulated in comparison to that of the S2308 strain within macrophages. However, the *virB* promoter activity in wild-type strain S2308 did not stop at 8 h p.i. in this study, which may be due to different setting up of the time point in the cell infection assays.

On further study, we investigated the expression of *vjbR, blxR*, and *mdrA* genes that have been proven to regulate T4SS expression directly in the smooth *Brucella* strain. We found that *vjbR* expression was upregulated, and the expression of both *blxR* and *mdrA* were downregulated in the Δ*rfbE* mutant. The VjbR protein belongs to the LuxR family, a group of transcriptional regulators involved in the cell-to-cell communication process referred to as quorum sensing (QS), This process allows bacteria to sense changes in population density and coordinate adaptive responses, and acts as the main regulator of expression of the *virB* operon (Miller and Bassler, 2001; Uzureau et al., 2010; Weeks et al., 2010). A *vjbR* mutant of *B. melitensis* exhibits downregulated expression of both the *virB* operon and flagellar genes, either during vegetative growth or during intracellular infection, and is strongly attenuated in a mouse model of infection (Delrue et al., 2005). In addition, VjbR regulates exopolysaccharide synthesis or export, as well as the production of several outer membrane proteins, some of which are involved in virulence (Uzureau et al., 2007). In the present study, we found that deletion of *vjbR* in the Δ*rfbE* mutant significantly reduced its cytotoxicity in macrophages. The BlxR protein is the second QS-related regulator of *Brucella* that contains both the DNA- and AHL-binding domains characteristic of the LuxR-type proteins (Rambow-Larsen et al., 2008; Sieira, 2013). Deletion of *blxR* affects virulence and intracellular survival of *Brucella*, but to a lesser extent than deletion of *vjbR* (Rambow-Larsen et al., 2008). A previous report suggests that BlxR negatively modulates activity of the *virB* promoter in *B. abortus* (Caswell et al., 2012). Our results confirmed that BlxR negatively modulates the activity of the *virB* promoter in *B. abortus*, and overexpression of *blxR* in the Δ*rfbE* mutant reduces to some extent, the cytotoxicity within macrophages. However, overexpression of *mdrA* in the Δ*rfbE* mutant did not reduce cytotoxicity within macrophages. The significance of *mdrA* downregulation in the Δ*rfbE* mutant requires further study. Thus, it is evident that a QS-related transcriptional regulator plays important roles in *Brucella* rough mutant-induced macrophage death. The QS-related transcriptional regulators might function in sensing environmental changes, such as cell density, acidification, and nutritional deprivation. One possible explanation is that loss of LPS in *Brucella* makes it sensitive to environmental stress that dysregulates the QS-related transcriptional regulators and upregulates T4SS to secrete a greater number of effectors. This probably accounts for the cytotoxicity in macrophages infected by *Brucella* rough mutants.

During *Brucella* interaction with host cells, the *Brucella* T4SS effector protein VceC, is involved in the induction of inflammatory responses by binding chaperone BiP, to trigger ER stress (de Jong et al., 2013). The ER stress induces the UPR in macrophages, and activates IRE1α, which in turn, recruits the NOD-like receptors NOD1 and NOD2, to induce activation of NF-κB and expression of pro-inflammatory genes (Keestra-Gounder et al., 2016). *Brucella abortus* inhibits cell death of infected macrophages (Fernandez-Prada et al., 2003; He et al., 2006), and chronically persists under conditions of a mild inflammatory response that leads to granuloma formation (Silva et al., 2011). However, the ER stress sensor IRE1α, induced by the rough mutant RB51, induces ROS-dependent NLRP3 translocation to mitochondria, and NLRP3 stimulates the caspase-2-Bid mitochondrial damage pathway, thereby leading to the release of mitochondrial danger signals that activate the inflammasome (Bronner et al., 2015). In this study, we found that the rough mutant Δ*rfbE*, secreted more effector proteins and induced stronger IRE1α pathways of ER stress, in comparison to the smooth wild-type strain S2308. These actions might excessively activate the IRE1α pathway and further activate the inflammasome via NLRP3- and caspase-2- driven mitochondrial damage, and result in cell death of macrophages. The crucial components associated with activation of the IRE1α pathway of ER stress to promote macrophage death in rough mutants remain to be identified.

Taken together, this study provided evidence that VjbR upregulation in the *Brucella* rough mutant Δ*rfbE* increases transcription of the *virB* operon, resulting in T4SS overexpression, accompanied by over-secretion of T4SS effector proteins. This in turn, strongly activates the IRE1α pathway of ER stress to cause the death of infected macrophages. This study provides novel insights into molecular mechanisms of *Brucella* rough mutant Δ*rfbE*-induced macrophage cytotoxicity.

## Author contributions

SY, MT, and CD conceived and designed the experiments; PL and MT mainly performed the experiments and analyzed the data; YB, HH, JL, YY, and SW helped to perform some experiments; PL wrote the paper, SY revised the manuscript and coordinated the research. All authors have read and approved the manuscript.

### Conflict of interest statement

The authors declare that the research was conducted in the absence of any commercial or financial relationships that could be construed as a potential conflict of interest.
